# Elucidating the Role of InGaAs and InAlAs Buffers on Carrier Dynamics of Tensile-Strained Ge Double Heterostructures

**DOI:** 10.1021/acsaelm.4c00347

**Published:** 2024-06-06

**Authors:** Shuvodip Bhattacharya, Steven W. Johnston, Robert J. Bodnar, Mantu K. Hudait

**Affiliations:** † Advanced Devices & Sustainable Energy Laboratory (ADSEL), Bradley Department of Electrical and Computer Engineering, Virginia Tech, Blacksburg, Virginia 24061, United States; ‡ National Renewable Energy Laboratory, Golden, Colorado 80401, United States; § Fluids Research Laboratory, Department of Geosciences, Virginia Tech, Blacksburg, Virginia 24061, United States

**Keywords:** germanium, carrier lifetime, tensile strain, molecular beam epitaxy, X-ray diffraction, photoconductance, bulk lifetime, surface recombination velocity

## Abstract

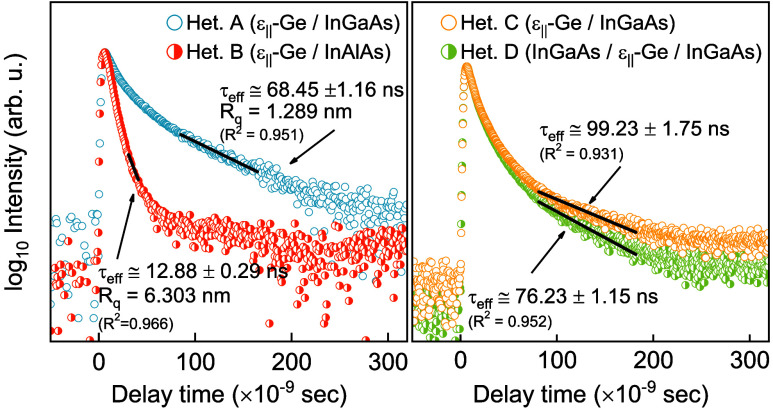

Extensive research efforts of strained germanium (Ge) are currently underway due to its unique properties, namely, (i) possibility of band gap and strain engineering to achieve a direct band gap, thus exhibiting superior radiative properties, and (ii) higher electron and hole mobilities than Si for upcoming technology nodes. Realizing lasing structures is vital to leveraging the benefits of tensile-strained Ge (ε-Ge). Here, we use a combination of different analytical tools to elucidate the effect of the underlying InGaAs/InAlAs and InGaAs overlaying heterostructures on the material quality and strain state of ε-Ge grown by molecular beam epitaxy. Using X-ray analysis, we show the constancy of tensile strain in sub-50 nm ε-Ge in a quantum-well (QW) heterostructure. Further, effective carrier lifetime using photoconductive decay as a function of buffer type exhibited a high (low) defect-limited carrier lifetime of ∼68 ns (∼13 ns) in 0.61% (0.66%) ε-Ge grown on an InGaAs (InAlAs) buffer. These results correspond well with the measured surface roughness of 1.289 nm (6.303 nm), consistent with the surface effect of the ε-Ge/III–V heterointerface. Furthermore, a reasonably high effective lifetime of ∼78 ns is demonstrated in a QW of ∼30 nm 1.6% ε-Ge, a moderate reduction from ∼99 ns in uncapped ε-Ge, alluding to the surface effect of the overlying heterointerface. Thus, the above results highlight the prime quality of ε-Ge that can be achieved via III–V heteroepitaxy and paves a path for integrated Ge photonics.

## 1 Introduction

The recent resurgence of interest in germanium (Ge) in the academic, technological, and commercial communities can be attributed to its unique properties that can be applied to electronic and photonic applications.^1,2^ One such attractive property is the potential to use band gap engineering to achieve direct band gap photoluminescence in Ge.^3−5^ In addition, Ge is compatible with the existing manufacturing infrastructure for silicon (Si), making it a popular alternative material for Si-compatible photonic applications.^6^ In fact, introduced at the 90 nm node, Ge has since been alloyed with Si in the drain/source well regions to impart uniaxial strain to the Si channel region to improve channel mobility.^7,8^ In a significant development, Liu et al. reported optical gain at room temperature in Ge directly grown on Si.^9^ This was made possible by leveraging the nominal ∼0.2% tensile strain due to the difference in thermal expansion coefficients and heavy n-type doping. With the increasing need for optical alternatives for intra- and interchip communication following the saturation of performance increases in traditional copper interconnects,^10−12^ Ge offers to bridge the gap with its improved radiative recombination efficiency.^13,14^ On the other hand, the concurrent rise in demand for high-mobility channel materials to enhance logic performance in the approaching sub-3 nm technology nodes, which exceeds the capabilities of Si, is encouraging increased interest in Ge due to its intrinsically higher electron and hole mobilities than Si.^1,6^

Due to the pseudodirect band gap of Ge, researchers have employed several different methodologies to enhance the radiative efficiency via strain engineering,^15−17^ band-structure modification,^18,19^ Sn-alloying,^20^ innovative structures for lasing in the form of nanomembranes,^21,22^ microcavities^23^ and microdisks,^24−26^ and more recently, direct epitaxial Ge growth on In_0.52_Al_0.48_As lattice-matched to InP,^27^ resulting in the formation of self-assembled Ge quantum dots (QDs). However, despite the reported success of some of these techniques to enhance radiative emission in Ge and Ge-based materials, most of these techniques suffer from incompatibility with the development of group IV-based lasing structures due to the lack of tunability of the electronic and optical confinement and/or due to complex microfabrication or growth processes, hindering large-scale integrability. On the other hand, one widely researched strategy for integration of Ge on Si has been using III–V metamorphic buffers, which can yield lower dislocation densities and improved crystalline quality.^28,29^ The concomitant benefit of utilizing III–V metamorphic buffers is the flexibility of tuning the underlying lattice parameters to impart strain, tensile and compressive, to the active Ge epilayer to achieve strain and band gap engineering, as well as providing tunability of optical confinement.^29−31^ Ternary (Ga, Al)-InAs-based buffers have been effectively utilized in the epitaxial growth of Ge for this purpose on on-axis and vicinal Si and III–V substrates. It has been shown previously that thick GaAs grown directly on Si can be used to block propagating threading dislocations from reaching the surface,^32,33^ which can then act as the starting surface for growing epitaxial films with high crystalline quality. Maximizing the potential of strain and band gap engineering of Ge for photonic applications requires the material system to be compatible with the development of quantum well (QW) heterostructures.^34^ Ideally, this entails including a cladding layer on either side with a large band gap and optical refractive indices distinct from the active layer for appropriate optical gain and optical confinement, respectively. That said, the present literature lacks the effect of the dual heterointerfaces in such QW heterostructures on the material properties and carrier recombination dynamics of strain-engineered Ge.

Therefore, in this work, we have undertaken a comparative analysis of the structural properties and carrier dynamics of pseudomorphic tensile-strained Ge (ε-Ge) integrated on vicinal (001)GaAs substrates via III–V buffers. Metamorphic InGaAs and InAlAs buffers grown using solid source molecular beam epitaxy (MBE) were used to impart strain to the overlying epitaxial Ge, and our results demonstrate pseudomorphic tensile-strained Ge epitaxy, verified by high-resolution X-ray diffraction analyses. We present a detailed characterization of the relaxation dynamics in the buffers and its effect on the surface morphology of ε-Ge using atomic force microscopy and X-ray analyses. Furthermore, we demonstrate the constancy of Ge strain state post overlayer growth on the top, a structure that emulates a practical QW lasing heterostructure. Finally, we demonstrate the reasonably high defect-limited effective carrier lifetimes achieved in InGaAs/ε-Ge/InGaAs and ε-Ge/InGaAs heterostructures compared to those of ε-Ge/InAlAs, simultaneously providing substantial evidence for the viability of strain-engineered Ge-based optical sources and photonic devices.

## 2 Materials and Methods

### 2.1 Material Synthesis

**Figure 1:**
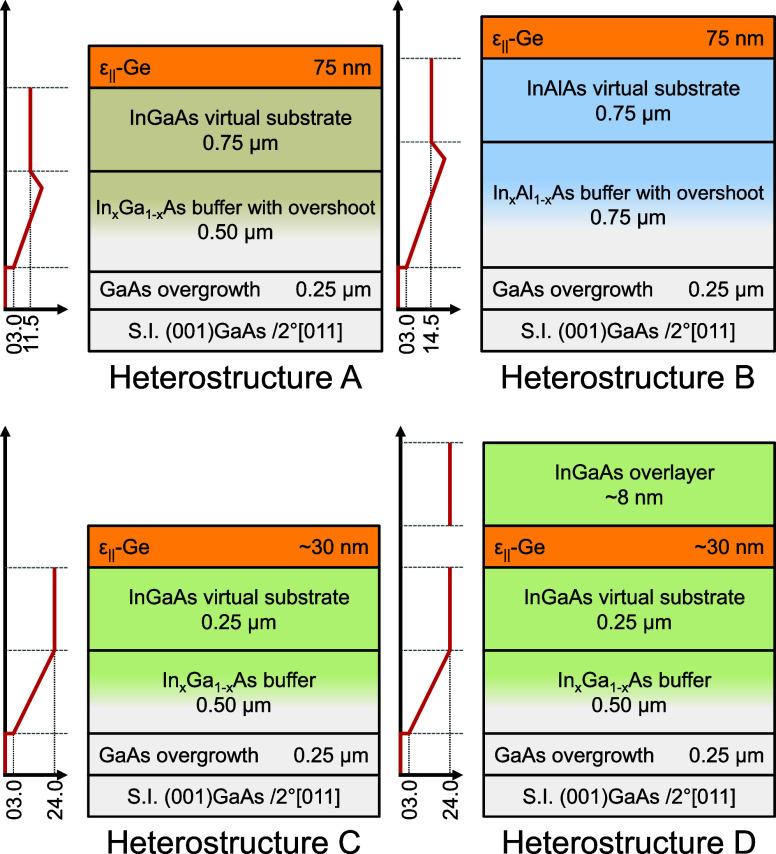
Representative schematics of the heterostructures studied in this work. Toward the left of each heterostructure, the InAs composition grading (solid red) with growth thickness is represented, along with the estimated compositions from XRD analyses on the abscissa. The figure is not drawn to the scale.

The heterostructures studied in this work, shown in Figure 1, were grown using solid source MBE using isolated group III–V and group IV chambers, connected via an ultrahigh vacuum chamber. Linearly graded metamorphic In_*x*_Ga_1–*x*_As and In_*x*_Al_1–*x*_As buffers were grown on epi-ready semi-insulating vicinal (001)GaAs/2°[011] substrates due to their ability to promote enhanced relaxation as compared to step-graded and nonlinear buffers.^35^ The isolation of the growth chambers minimizes the possibility of atomic interdiffusion at the group IV/group III–V heterointerfaces during epitaxial growth. Each growth run was monitored using an *in situ* reflection high-energy electron diffraction (RHEED) module inside the III–V growth chamber. Oxide desorption of each substrate was performed at (thermocouple temperature) ∼750 °C, maintaining a high arsenic (As_2_) overpressure of ∼10^–5^ Torr to prevent degradation of III–V surface morphology. As such, subsequent growth was performed only after observation of long and clear (2 × 4) patterns on the RHEED screen, suggesting good oxide desorption. Linearly graded metamorphic In_*x*_Ga_1–*x*_As and In_*x*_Al_1–*x*_As buffers in heterostructures A and B were grown at 525 and 420 °C, respectively, with the lower temperature used for the latter to balance the disparate adatom surface mobilities of indium (In) and aluminum (Al) as well as a lower growth rate, with an additional overshoot and inverse step to promote enhanced relaxation of the buffers. Linearly graded In_*x*_Ga_1–*x*_As buffers for heterostructures C and D were grown at 550 °C with no overshoot. A growth pause and an additional annealing step at 540 °C were used for heterostructure B to enhance relaxation in the low-temperature grown buffer.^36^ Both constant composition In_0.115_Ga_0.885_As and In_0.145_Al_0.855_As virtual substrates in heterostructures A and B were grown at 525 °C, whereas In_0.24_Ga_0.76_As virtual substrates in heterostructures C and D were grown at 550 °C. Following immediate transfer to the group IV chamber, unintentionally doped epitaxial Ge was grown at 400 °C at a nominal growth rate of ∼0.067 Å/s. For heterostructure D, a constant composition overlayer of In_0.24_Ga_0.76_As was grown at 400 °C to emulate a QW heterostructure, where a lower temperature was used to prevent strain relaxation in the Ge epilayer.

### 2.2 Material Characterization

Crystallinity, relaxation of the buffers, InAs composition in the constant composition virtual substrates, and strain state of the epitaxial Ge layers were studied using high-resolution X-ray diffraction (HR-XRD). *Ex situ* symmetric (004) and asymmetric (115) reciprocal space maps and symmetric (004) rocking scans in the triple axis configuration were measured from each heterostructure using a PANalytical X’Pert Pro diffractometer equipped with PIXcel and proportional detectors, with a monochromatic Cu Kα_1_ (λ = 1.540597 Å) X-ray source. Additional tensile strain estimation of epitaxial Ge layers was done using Raman spectroscopy in the backscattering (001) geometry on a JY Horiba LabRam HR800 system equipped with a 514.32 nm Ar^+^ laser source, and 1800 lines/mm gratings were used during measurement. The laser power at the sample surface was ∼10 mW. The surface morphology of the heterostructures was studied using Bruker Dimension Icon atomic force microscopy (AFM) configured in the tapping mode. Subsequent stage tilt correction during AFM scans was performed using the native Nanoscope Analysis software package included in the Bruker AFM ecosystem. Finally, the effective carrier lifetime of the strain-engineered Ge epilayers was measured at room temperature (∼300 K) using a conventional microwave-reflectance photoconductive decay (μ-PCD) system at the National Renewable Energy Laboratory (NREL). Representative cleaved pieces of 1 cm × 1 cm from each heterostructure were placed under a waveguide (WR42 at 20 GHz, 0.43 cm × 1.07 cm) and optically excited using a Q-switched neodymium-doped yttrium aluminum garnet (Nd/YAG) laser. A wavelength (λ) of 1500 nm was chosen for the excitation to ensure homogeneous excitation and photocarrier generation in the Ge epilayers. A nominal optical power of 20 mW was used, as measured by a power meter with an absorption disk diameter of 2 cm. A repetition rate of 10 pulses per second and a pulse width of 5 ns were used. With the above, this resulted in a low injection level of ∼10^14^ cm^–3^ s^–1^, calculated from the effective photon flux of ∼10^17^ s^–1^.

## 3 Results and Discussion

### 3.1 Analysis of Strain State and Composition Using HR-XRD

The schematics of the heterostructures investigated in this work are shown in Figure 1. Detailed crystalline and structural analysis was conducted on the heterostructures using HR-XRD measurements. Typical symmetric (004) and asymmetric (115) reciprocal space maps (RSMs) were recorded from each heterostructure for quantification of the effective In incorporation in the virtual substrates and relaxation achieved in the buffer layers and final strain state and crystalline quality of the ε-Ge epilayers. We have reported XRD measurements from heterostructure A in our previous work,^37^ and they are reported here to aid in direct comparison with heterostructure B.

#### 3.1.1 Cation (Ga, Al) Grading in an InAs-Based Buffer

**Figure 2:**
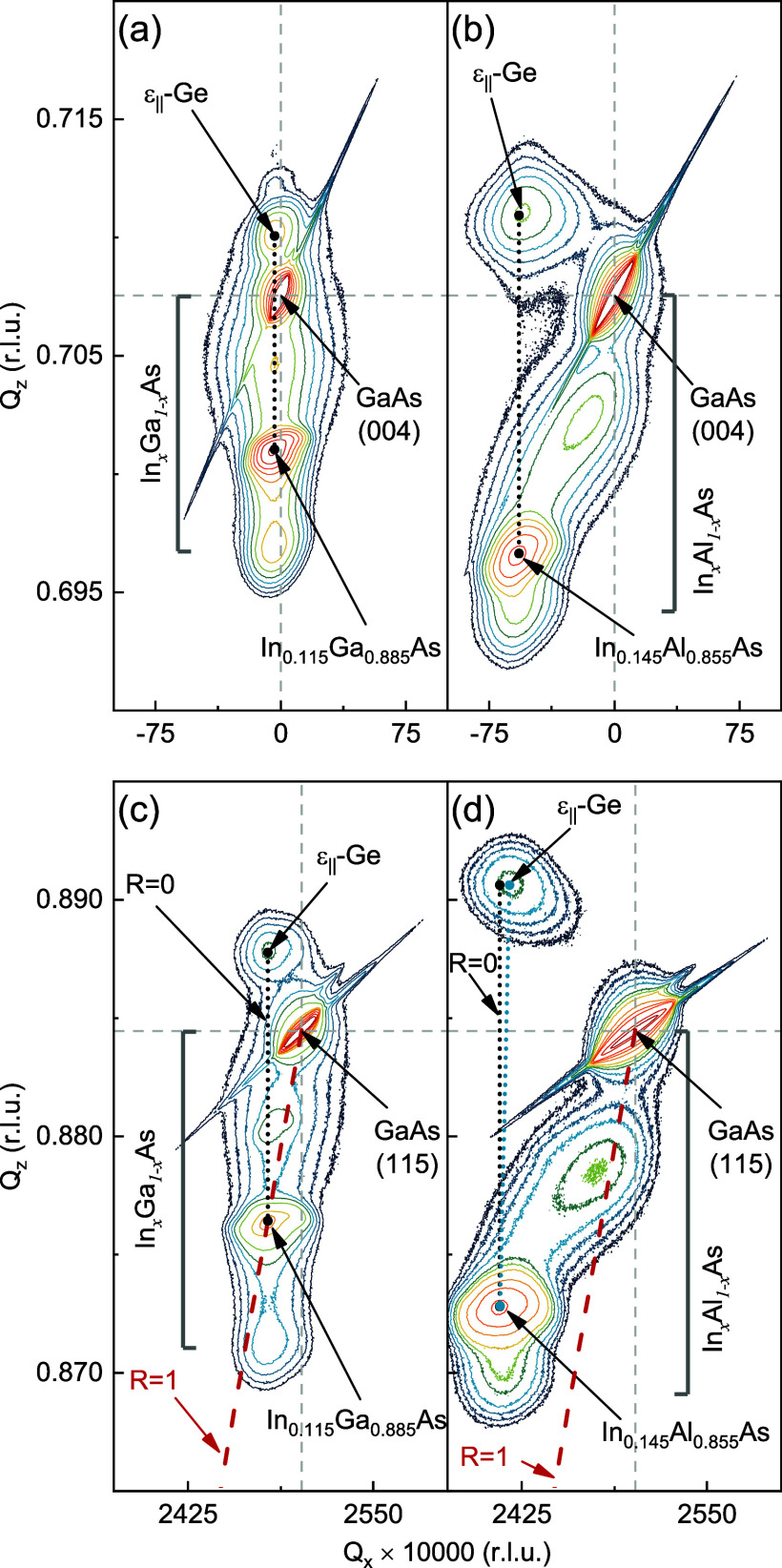
(a, b) Symmetric (004) RSMs obtained from heterostructures A^37^ and B, respectively. The alignment of the ε-Ge RLCs (black dotted) with the InGaAs and InAlAs virtual substrates confirms pseudomorphic epitaxy. (c, d) Asymmetric (115) RSMs obtained from heterostructures A^37^ and B, respectively. The strained (dotted black) and relaxed (dashed red) lines are shown. The slight misalignment of the ε-Ge RLCs with the InAlAs RLC is indicated (dotted blue).

Plotted in reciprocal space coordinates, symmetric (004) RSMs recorded from heterostructures A and B are shown in Figure 2a,b, respectively. For a symmetric (004) RSM, the iso-intensity contours from all epilayers should be aligned along the *Q*_*x*_ axis in the absence of tilt or other finite crystal effects, as only the out-of-plane lattice parameter (*a*_⊥_) is probed in this configuration. The reciprocal lattice contour centroids (RLCs) from the different epilayers would be displaced up or down while being aligned along this axis. Figure 2a,b shows a few important characteristics and distinctions between heterostructures A and B. First, the RLCs from the ε-Ge epilayers are vertically displaced above the GaAs RLCs in both heterostructures, suggesting a compressed out-of-plane lattice parameter, *a*_⊥_, and stretched in-plane lattice parameter, *a*_∥_, indicating successful transfer of tensile strain from the underlying buffer layers to the Ge layer. Further, the difference in vertical position of the ε-Ge iso-intensity RLCs in the heterostructures indicates a slightly higher tensile-strained Ge in heterostructure B than in heterostructure A. Another important observation is the difference in deviation of the epilayer RLCs from the *Q*_*x*_(00*l*) = 0 line; larger deviations indicate a larger amount of tilting with respect to the substrate orientation present in the epilayers. In this case, much larger tilting is observed in heterostructure B. This observation can shed light on the relaxation dynamics originating during growth of the metamorphic buffers, which will be explored shortly. Finally, below the constant composition InGaAs and InAlAs RLCs, the observable RLCs are contributions from the overshoot composition layers incorporated to promote increased relaxation of the linearly graded buffers.

Since symmetric (004) reciprocal space maps yield information solely about the lattice spacing in the direction of growth, asymmetric (115) reciprocal space maps were also recorded from heterostructures A and B to obtain insight into the in-plane lattice spacing, *a*_∥_. These scans are shown in Figure 2c,d. The fully relaxed (*R* = 1 in dashed red, denoting 100% relaxation) reference line is indicated as well. The RLCs of the individual epilayers should align along this line if the epilayers are fully relaxed and not tilted with respect to the substrate. Additionally, the fully strained line (*R* = 0 dotted black, denoting 100% strained) is indicated as well to denote the vertical alignment of the Ge RLCs to the constant composition virtual substrate RLCs in each heterostructure. Due to the low angle of incidence used in an asymmetric scan, the corresponding RLCs of the epilayers are split further apart in the reciprocal space. In heterostructure A, the constant composition InGaAs virtual substrate RLC lies near the fully relaxed line, implying a high degree of symmetric relaxation. On the other hand, the large deviation of the constant composition InAlAs virtual substrate RLC in heterostructure B from the fully relaxed line suggests partial and/or asymmetric relaxation and the presence of significant tilt in the film. However, it is important to note that the Ge epilayer RLC in heterostructure A is well aligned with the constant composition InGaAs virtual substrate RLCs along the *Q*_*x*_ axis, indicating that the Ge epilayer is pseudomorphically strained. We note that a minor misalignment of the Ge epilayer with respect to the constant composition InAlAs virtual substrate in heterostructure B is present, as illustrated by the dotted blue line connecting the epilayer RLCs. The symmetric and confined nature of the RLCs of the constant composition virtual substrates indicates that defects generated from mismatched heteroepitaxy were generally contained within the linearly graded metamorphic buffers, thereby decreasing the propagation of dislocations to the Ge epilayers. Following the methodology outlined in refs (38) and (39), comprehensive quantification of the InAs molar fraction true to Vegard’s law, residual strain in each constant composition virtual substrate, and tensile strain imparted to each Ge epilayer is carried out and is reported in Table 1. The tensile strain values for the Ge epilayers are determined to be 0.61 ± 0.05 and 0.66 ± 0.05%, respectively, for heterostructures A and B, with the error being linked with finding the peak centroid for the corresponding epilayers. This agrees with the slightly larger vertical displacement of the Ge RLCs in heterostructure B compared with heterostructure A. Nominal InAs concentrations of 11.5 and 14.5% are obtained from the constant composition InGaAs and InAlAs layers, respectively, which is again in line with the larger vertical displacement of the constant composition layer RLCs. The InAlAs constant composition virtual substrate, however, shows a higher degree of residual strain compared with the InGaAs constant composition virtual substrate. A moderately low tilt of ∼142 arcsec is observed in heterostructure A, suggesting a symmetric buffer relaxation process during growth. The minor tilt is possibly due to the asymmetry originating from the difference in nucleation energy and glide velocity of group V-terminated α (along ⟨11̅0⟩) and group III-terminated β (along ⟨110⟩) dislocations.^40,41^ On the other hand, the observed tilt of ∼1600 arcsec coupled with the high residual strain within the InAlAs metamorphic buffer suggests asymmetric strain relaxation and a large magnitude of misfit dislocations with their Burgers vector aligned normal to the substrate orientation, resulting in tilted epitaxy. Ternary In_*x*_Al_1–*x*_As buffers are challenging to grow due to the large differences in adatom surface mobilities between Al and In, which naturally lends itself to an increased difference in preferred nucleation and subsequent glide of α and β dislocations. Additionally, this disparity can lead to asymmetrical strain relaxation and therefore result in lattice tilt, as is observed in the case of heterostructure B.^41^ It is important to note that the presence of tilt can drastically distort the accurate measurement of strain state in the Ge epilayers via XRD. Thus, we have further assessed the strain in the Ge epilayers using Raman spectroscopy, as discussed below.

**Table 1:** Summary of InAs Composition, Epilayer Tilt, and Tensile Strain State in ε-Ge Estimated from XRD and Raman Analyses from Heterostructures A and B

		lattice parameters (Å)						tensile strain, ε_∥_ (%)		
het.	layer	*a*_⊥_	*a*_∥_	*a*_relaxed_	InAs molar fraction (%)	epilayer tilt (arcsec)	relaxation (%)	XRD	Raman	theory
A^37^	InGaAs	5.7065	5.6924	5.6998	11.5	–142	84			
	ε-Ge	5.6329	5.6864	5.6597		–115		0.61 ± 0.05	0.77 ± 0.08	0.74
B	InAlAs	5.7424	5.6954	5.7189	14.5	–1610	64			
	ε-Ge	5.6253	5.6910	5.6581		–1502		0.66 ± 0.05	0.82 ± 0.08	1.07

#### 3.1.2 Overlayer Growth and Strain State in (001) Biaxially Tensile-Strained Ge

**Figure 3:**
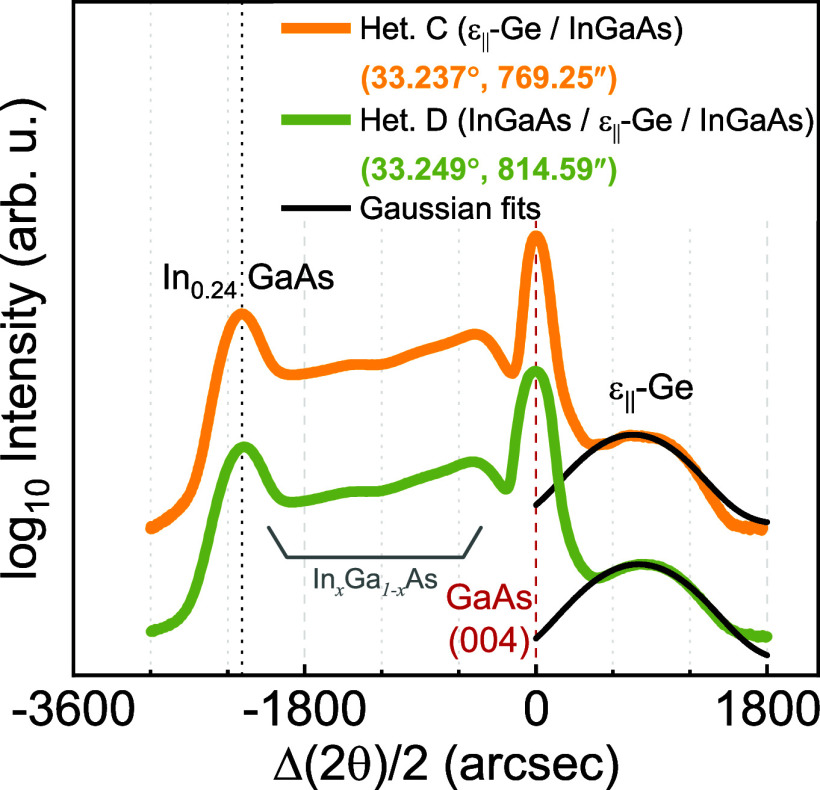
Projection of symmetric (004) RSM spectra to the Δθ =Δ(2θ)/2 axis recorded from heterostructures C and D. The peak positions of the ε-Ge epilayers were found using weighted Gaussian fits to the projection spectra. The Bragg peak positions (°) and full width at half-maximum (fwhm) (arcsec, ″) values obtained from the fits are included in the legend box.

As noted earlier, to actualize waveguides for the potential of optical communication, QW heterostructures are necessary so that the tensile strain-engineered Ge epilayers are embedded between III–V cladding layers.^34^ It is crucial to ensure that the Ge epilayer does not relax during the growth of the overlying layers. Therefore, to complement and compare to our previously published work,^42^ we have grown an InGaAs capping overlayer on top of the Ge epilayer to realize the QW heterostructure. Symmetric (004) RSMs were recorded from heterostructures C and D, and the projection of the recorded intensities onto the Δθ axis is shown in Figure 3. Here, Δθ = Δ(2θ)/2 and is the angular displacement of the epilayers with respect to the substrate, determined by their peak positions measured in scattering angle, 2θ. This way, the contributions to the contours from the different epilayers and the subsequent differences in peak positions can be directly observed and compared without the effect of tilt. We emphasize here that a lower temperature was utilized for growing the In_0.24_Ga_0.76_As overlayer to ensure that the Ge epilayer did not relax during overlayer growth. As shown in Figure 3, the tensile-strained Ge peaks from heterostructures C and D lie on the higher diffraction angles to the right of the GaAs substrate peak, in accordance with the expansion (compression) of the *a*_∥_ (*a*_⊥_) lattice parameter because of the tensile strain imparted from the underlying buffer. The peak positions of the ε-Ge epilayers with respect to the GaAs substrate peak position (758.13108 arcsec (absolute 33.237°) and 800.69156 arcsec (absolute 33.249°), respectively, from heterostructures C and D) were determined by using weighted Gaussian fits. A minimal difference of 42.56 arcsec in the Ge peak positions was observed, which results in |Δ*a*_⊥, Het. C–Het. D_| = 0.00176 Å, indicating virtually no relaxation of the ε-Ge epilayer in heterostructure D, while emphasizing the difference could stem from minor variations in InAs incorporation during growth of the InGaAs virtual substrates. Given that the strain state in the Ge epilayer remains unaltered post overlayer growth, one can expect coherent top and bottom heterointerfaces and an essentially defect-free active ε-Ge epilayer. This marks a significant result in that strain-engineered Ge QW heterostructures with tensile strains beyond the crossover threshold^43^ can be developed while maintaining prime material quality.

### 3.2 Strain State Estimation Using Raman Spectroscopy

In addition to diffraction investigation, we employed Raman spectroscopy to measure the level of tensile strain in the Ge epilayers in heterostructures A and B. The effect of biaxial strain on phonon modes is extensively elucidated by Sui and Herman.^44^ Essentially, biaxial strain in the (001) plane in diamond-cubic crystals causes the triply degenerate optical phonon modes at the center of the Brillouin zone to be split into a singlet and a doublet with the eigenvectors perpendicular and parallel to the (001) plane, respectively. With the degeneracy lifted, an upshift (downshift) in the angular frequency of the singlet (doublet) is observed. In the (001) backscattering mode normally utilized in Raman spectroscopy, scattering from the doublet is forbidden by the principles of symmetry. As such, only the long-wavelength longitudinal optical (LO) mode is the active Raman mode that can be detected in this geometry. In addition, epilayer strain induces a shift of the singlet LO phonon frequency (Ω_s_) and, consequently, a shift in the Raman frequency is observed. Accordingly, this frequency shift in the Raman active mode (Δω) because of (001) biaxial stress can be expressed in terms of stress tensors, σ_*xx*_ and σ_*yy*_, and material-specific elastic compliance tensor components *S*_*ij*_, as follows:
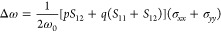
1

**Figure 4:**
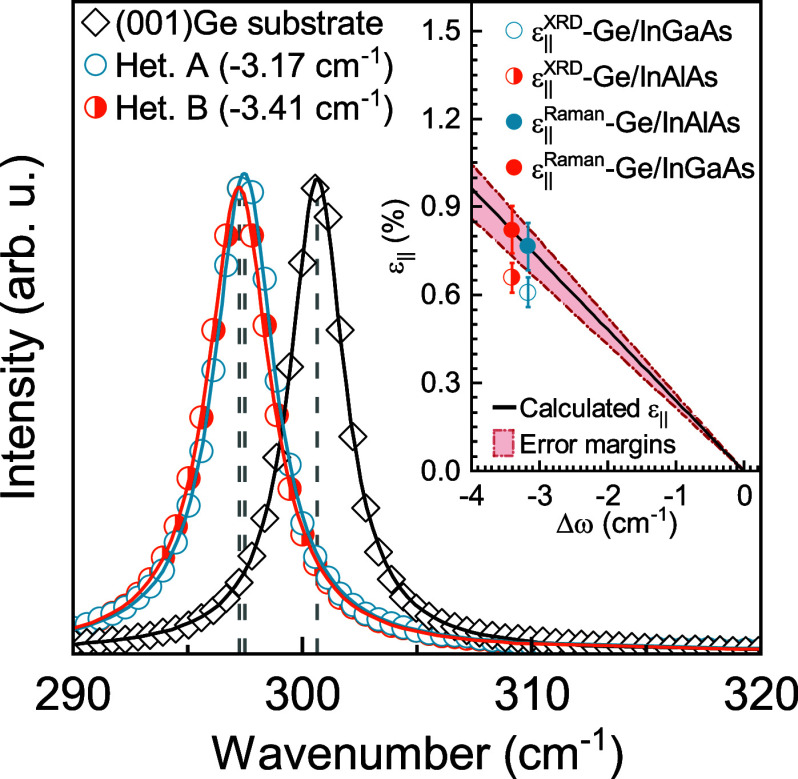
Raman spectra obtained from heterostructures A and B overlapped with Raman spectra recorded from the (001)Ge substrate. Peak positions were found using Lorentzian fits to the spectra. The inset shows the strain relationship between the recorded Raman spectra and the tensile strain calculated using Δω = *b*·ε_∥_, where *b* and ε_∥_ denote the fitting parameter and in-plane strain, respectively. The error margins for strain estimation are indicated by the shaded red regions. In-plane tensile strains estimated from XRD (Table 1) are also indicated by the corresponding symbols.

Here, *p* and *q* are the optical phonon deformation potentials, and ω_0_ is the frequency of the active Raman mode in the absence of mechanical stress. The relation can be further simplified to Δω = *b*ε_∥_, given that σ_*xx*_ = σ_*yy*_ in (001) biaxial stress and that in such a formalism, the Raman shift is linear with strain. Here, *b* = (*q* – *p*(*C*_12_/*C*_11_))/ω_0_, where *C*_*ij*_ are material-specific elastic constants and ε_∥_ indicates in-plane strain. Such a simplification is made feasible under the assumption of a biisotropic tetragonal strain, which is the case in this study. Using material-specific parameters from ref (45), Fang et al.^46^ calculated *b* = −415 ± 40 cm^–1^. Hence, a down shift (upshift) of the frequency of the Raman active mode should indicate the presence of tensile (compressive) strain within the material system. Figure 4 shows the measured Raman spectra obtained from heterostructures A and B, overlapped with the Raman spectra obtained from the (001) Ge substrate. Relative active Raman mode wavenumber offsets of −3.18 and −3.41 cm^–1^ were obtained with respect to the (001) Ge substrate active Raman mode, respectively, from the Ge epilayers on heterostructures A and B. Accordingly, the effective tensile strain in the Ge epilayers were inferred to be 0.77 ± 0.08 and 0.82 ± 0.08%, respectively. The term “effective” is used here to emphasize the difference in the absolute values of strain estimated from XRD and Raman spectroscopy. The inset of Figure 4 demonstrates the relationship between the measured relative shift and subsequently calculated tensile strain and the estimated strain from XRD analysis. The shaded region represents the limits in estimates of strain from Raman spectroscopy arising from the uncertainty in the value of *b*, which is the fitting parameter. The results are also summarized in Table 1. As mentioned earlier, stemming from the anisotropic nature and different pathways of strain relaxation in the In_*x*_Ga_1–*x*_As and In_*x*_Al_1–*x*_As metamorphic buffers, epilayer tilt might play a significant role in obscuring the accurate representation of measured strain via XRD. Since optical phonon modes contain information solely about the bond angles and bond lengths and, in the presence of mechanical strain, bond angles and lengths are deformed, one could argue that relative shifts in Raman active modes might give a more realistic representation of strain in the epilayers. Considering fully relaxed constant composition InGaAs and InAlAs, respectively, with InAs fractions of 11.5 and 14.5% as assessed from XRD (close to the target compositions for this work), the theoretical misfit can be derived to be ε_∥_ ≅ 0.74% and ε_∥_ ≅ 1.07%, respectively. This brings about an intriguing observation; the strain state inferred from Raman spectroscopy in heterostructure A is closer to the theoretical misfit but deviates significantly in the case of heterostructure B. Hoshina et al.^47^ reported a comparable disparity in strain measurements, between XRD and Raman spectroscopy, obtained from strained Ge grown on In_*x*_Ga_1–*x*_As metamorphic buffers. The authors observed that the deviation between XRD- and Raman-reported strain values was significantly higher in the high tensile strain region, starting around ∼1% (001) biaxial strain, and explained the phenomenological observation based on the gradual relaxation of the strained Ge epilayers grown on InGaAs metamorphic buffers with thicknesses exceeding the critical layer thickness. The Ge epilayers in heterostructures A and B in this work are grown well within the critical layer thickness, adhering to the strain balance model by People and Bean,^48^ and hence, the Ge epilayers should be fully strained. This is indeed observed in our previous work^37^ where high-magnification transmission electron micrographs obtained from the constant composition InGaAs virtual substrate, the ε-Ge epilayer, and the ε-Ge/InGaAs heterointerface show a high degree of coherence and absence of misfit dislocations, alluding to the pseudomorphic epitaxy of Ge. The exact cause for this observed disparity between XRD- and Raman-estimated strain levels, especially on InAlAs buffer, currently eludes the authors and needs additional research.

### 3.3 Surface Morphology Using AFM

The surface morphology of heterostructures A and B was examined using AFM since it can provide insight into the relaxation dynamics during growth. It is extensively documented that strain relaxation pathways in cubic (001)-oriented metamorphic buffers result in a crosshatch pattern on the surface.^49^ As the thickness of any strained heteroepitaxial film surpasses the critical thickness, the accumulated elastic strain energy transcends that of the thermodynamically permitted strain energy density, and the film undergoes plastic relaxation, thereby favoring the formation and subsequent glide of threading dislocations, which in turn form surface slip steps and segments of misfit dislocation at the heterointerface between the epilayer and the substrate. For the heteroepitaxy of (001)-oriented diamond or zinc blende cubic systems, the dominant class of slip systems is collectively referred to as ⟨110⟩{111}, where *a* is the lattice parameter;^50^ hence, such threading dislocations glide along the {111} slip planes in the ⟨110⟩ directions. Consequently, there are eight such combinations of ⟨110⟩{111} systems that can take part in strain relaxation. At the growth front, the surface slip steps lead to local surface roughness; lateral mass transport, i.e., movement of adatoms to preferred step sites, and step-flow growth processes are initiated to remove this locally developed roughness. The trailing misfit segments along the heterointerface take part in relaxing the stress in the epilayer, while local stress is produced at the dislocation cores. Eventually, hillocks and valleys are formed because of the strain relaxation process, which propagates along the dislocation lines near the heterointerface misfit dislocations. Several investigative works have reported on asymmetry in strain relaxation along the orthogonal ⟨110⟩ directions and its effect on the surface morphology. Another source of asymmetry, specifically in the case of low-mismatched compressive InGaAs heteroepitaxial growth on GaAs substrates, is the lower activation energy of dislocation nucleation and subsequent glide velocities for group V-terminated (α) dislocations than group III-terminated (β) dislocations, which are oriented along crystallographic [11̅0] and [110] directions, respectively. While this is equally true for InAlAs mismatched heteroepitaxy on GaAs, the large variation in surface adatom mobility between In and Al generally results in degraded surface morphology by virtue of phase separation and InAs and AlAs clustering.^51^

**Figure 5:**
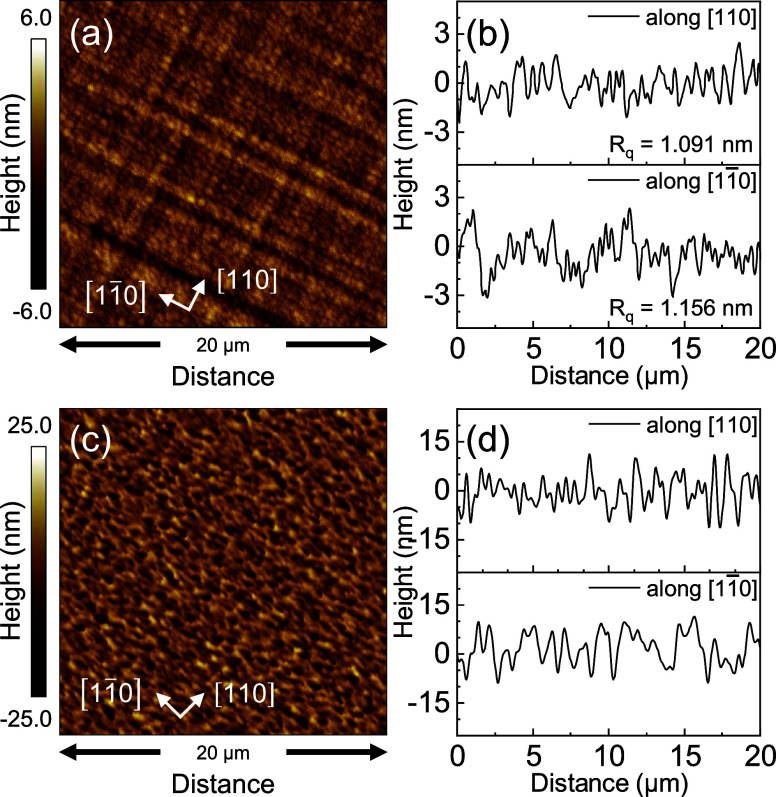
(a, c) Atomic force micrographs of 20 μm × 20 μm representative regions obtained from heterostructures A and B, respectively. The orthogonal directions ⟨110⟩ are indicated. (b, d) Line profile scans showing the height variations along the orthogonal directions. Orthogonal *R*_q_ values for heterostructure B are not reported in panel (d) due to the absence of an observable cross-hatch.

AFM micrographs from heterostructures A and B are shown in Figure 5. Heterostructure A, incorporating the InGaAs metamorphic buffer, exhibits a characteristic symmetric crosshatch pattern. The development of the cross-hatch pattern suggests a rather symmetrical relaxation process during the growth of the metamorphic buffer, as all eight slip systems seem to have contributed equally. This observation is further corroborated by the similar root-mean-squared roughness (*R*_q_) values of 1.156 and 1.091 nm along the orthogonal [11̅0] and [110] directions, respectively. Consequently, the representative region shown exhibits a smooth surface with a modest *R*_q_ value of 1.289 nm. In contrast, heterostructure B, with the InAlAs metamorphic buffer, displays a randomized rough pattern with a significantly higher *R*_q_ value of 6.303 nm. No clear crosshatch pattern is visible in the representative region. The strain relaxation process in mismatched heteroepitaxy is greatly impacted by the growth temperature and the disparity in adatom surface mobilities. For heterostructure A, a growth temperature of 525° was possible due to the similar adatom surface mobilities of In and gallium (Ga). On the other hand, to minimize the difference in adatom surface mobility between In and Al, a lower temperature of ∼420 °C was chosen for the growth of the InAlAs linearly graded metamorphic buffer. A subsequent *in situ* annealing step was performed at a higher temperature of 540 °C for a duration of 15 min prior to the growth of the constant composition InAlAs layer at 525 °C. The purpose of this annealing step was to promote relaxation of the linearly graded buffer and preferably reduce the tilted components typically observed in low-temperature buffer growths.^36^ These results are consistent with the findings of Chyi et al.,^52^ where the authors found a rougher surface with linearly or step-graded InAlAs buffers grown in the range of 420–520 °C than all variations of growth of InGaAs in the same temperature range and similar In composition. Given the above low relaxation of the buffer and slightly higher substrate misorientation estimated from XRD analyses, the higher surface roughness in heterostructure B could be attributed to the combination of the following factors: (i) lower growth temperature, at which the surface mobility of Al adatoms is much lower than that of In, such that InAs- and AlAs-rich regions are created on the surface front, leading to local composition modulation and subsequent increase in surface roughness at the growth front, which accumulates over the growth thickness; (ii) the slightly larger GaAs substrate misorientation, determined from X-ray analysis, which leads to additional surface steps^53^ and consequently leads to variable critical resolved shear stress for the eight slip systems, with some of the planes energetically more favorable for nucleation and subsequent glide of dislocations and more active during strain relaxation;^40^ and finally, (iii) large disparity between, and a possible bias toward, nucleation and glide of α over β dislocations.^54^ It is well known that rough surfaces act as carrier trapping and recombination centers and that the surface plays a crucial role in carrier dynamics. In the next section, we investigated the effective carrier lifetimes of the ε-Ge epilayers in the different heterostructures using μ-PCD.

### 3.4 Effective Carrier Lifetime Analysis via μ-PCD

Material quality strongly affects the defect-limited carrier lifetime, which follows Shockley–Read–Hall (SRH) carrier dynamics and, as such, can indicate the viability of a material for device-based applications.^55,56^ The presence of defects and impurities within the bulk of the material can significantly alter the lifetime of carriers, as they act as trapping and recombination centers. Additionally, surface roughness plays a crucial role in adversely affecting the lifetime of carriers, with unpassivated active layers showing degraded carrier lifetime.^57^ The most widespread used technique in this regard is the μ-PCD technique, which allows for noncontact and rapid collection and analysis of minority carrier lifetime,^58−60^ circumventing multiple microfabrication steps that can alter the material quality. When a material is subjected to an optical source, excess photocarriers are generated inside of the material. Subsequently, when the optical source is removed, in response to the locally developed concentration gradient, these carriers start diffusing away from the area of high concentration and eventually recombine through several recombination processes. This change in concentration in turn causes a change in the local conductance of the material, which can be recorded using a microwave source probe and thus provides information about the carrier dynamics. Depending on the choice of the wavelength used, one can also independently probe different parts of the heterostructure as well as probe the bulk recombination processes that dominate the lifetime far away from the surface.^61^ For this work, we used the μ-PCD technique to characterize the effect of the underlying buffer and top overgrowth on the effective carrier lifetime in the ε-Ge epilayers. Details of the measurement technique have been reported in our previous work.^37^ Under low-injection conditions, it can be shown that the reciprocal of effective carrier lifetime, τ_eff_, is expressed as the cumulative response from two reciprocal components, τ_surface_ and τ_bulk_, as follows:^62,63^
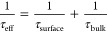
2where τ_surface_ and τ_bulk_ are effective lifetime components from the surface and bulk, respectively. Defects and other non-idealities within the bulk adversely affect the effective lifetime, and therefore, the effective minority carrier lifetime becomes bulk-limited. The same can be said for the surface component, and the effective lifetime then becomes surface-limited.

#### 3.4.1 Effect of the Strain Template on (001) Biaxially Tensile-Strained Ge

**Figure 6:**
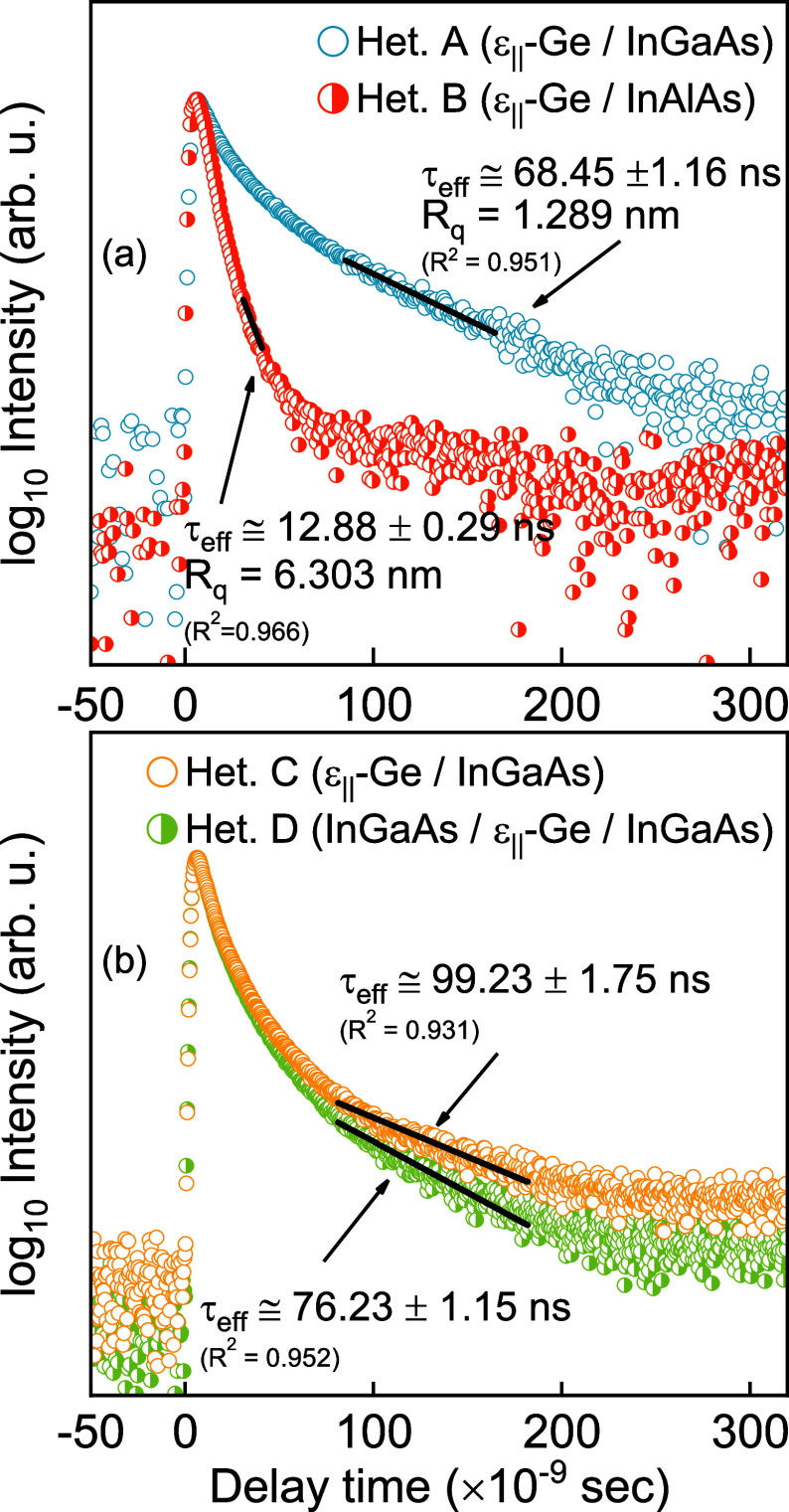
μ-PCD transient curves recorded from (a) heterostructures A^37^ and B and (b) heterostructures C and D, respectively. The effective lifetime is obtained in each case by fitting an exponential regression to the principal mode of decay (solid black), and the values along with the error and goodness of fit (*R*^2^) are labeled.

Figure 6a shows typical μ-PCD transients obtained from ε-Ge epilayers grown on heterostructures A and B. The absorption coefficient of Ge (α ≅ 2.5 × 10^4^ cm) at the excitation wavelength of λ = 1500 nm used ensures that the excess photocarriers are generated homogeneously throughout the Ge epilayer. Additionally, the underlying layers are transparent to the excitation at this wavelength, which ensures exclusive observation of the carrier dynamics within the Ge epilayers. The effective carrier lifetime, obtained by fitting an exponential decay regression to the principal mode of decay,^62^ is substantially higher in ε-Ge grown on the InGaAs metamorphic buffer (τ_eff_ ≅ 68 ns) compared to its counterpart grown on the InAlAs metamorphic buffer (τ_eff_ ≅ 13 ns). This observation can be explained as follows: when a semi-infinite crystal terminates on the surface, the pseudopotential energy on the surface is starkly different from that in the bulk of the material, where the periodicity of the crystal lattice is maintained. This termination leads to unsatisfied dangling bonds on the surface that can participate in recombination dynamics. Conversely, an increase in the surface roughness can be considered as an increase in the effective surface area and, thus, an increase in surface recombination centers due to the undulations. Thus, one can expect an increase in the rate of carrier recombination on the surface. In this case, when the optical source is removed, the generated photocarriers quickly diffuse toward the surface and recombine. While the thickness of the epilayer can also influence the effective lifetime, with longer effective lifetime typically observed in thicker epilayers, the Ge epilayers are of the same thickness in this work, and as such, thickness effects can be neglected. Therefore, it is modest to conclude that the observed difference in effective carrier lifetime is primarily due to the difference in surface recombination velocity; a higher surface recombination velocity results in reduced effective lifetime and vice versa. In view of the above discussion, the reduced effective lifetime in ε-Ge grown on the InAlAs metamorphic buffer corresponds well to our AFM findings and further corroborates the XRD results, wherein a high degree of incomplete strain relaxation and asymmetric relaxation led to a poor surface morphology. Likewise, the symmetric strain relaxation of the InGaAs metamorphic buffer resulted in a smoother surface morphology and leads to enhanced effective carrier lifetime in ε-Ge grown in heterostructure A.

#### 3.4.2 Effect of Overlayer Growth on (001) Biaxially Tensile-Strained Ge

We have further used μ-PCD to study the effect of the symmetric constant composition InGaAs overlayer on top of the ε-Ge epilayer. We previously noted in the XRD section that there was no apparent relaxation in the ε-Ge epilayer based on the positions of the diffraction peaks. Figure 6b shows typical PCD transients obtained from heterostructures C and D. The excitation wavelength for this case is kept at λ = 1500 nm for reasons cited above. As seen in Figure 6b, the modest but measurable reduction in effective carrier lifetime in heterostructure D (τ_eff, Het. D_ ≅ 76 ns, compared to τ_eff, Het. C_ ≅ 99 ns) could possibly be due to the inadequate surface passivation by the overlying InGaAs layer, the effect of low-temperature growth of constant composition InGaAs overlayer, resulting in a rougher heterointerface shared with the Ge epilayer, or the presence of electrically active antiphase boundaries between antiphase domains resulting from the growth of polar InGaAs on nonpolar Ge. This qualitative assumption is justified from the quicker decay of the transient curve in the capped sample, as the initial roll-off is exclusively dependent on the surface component^62^ of the lifetime expression in eq 2. Thus, following from the above discussion and the observation from Figure 3, it is modest to conclude that (i) the tensile strain in the Ge epilayer is not relaxed and (ii) the bulk lifetime in the ε-Ge epilayer is probably not affected by the overgrowth of the InGaAs epilayer and that the measurable reduction of the effective lifetime is a direct consequence of the surface properties of the top heterointerface. To the best of our knowledge, the carrier recombination dynamics of the ε-Ge QW heterostructure measured via μ-PCD has never been demonstrated earlier. Furthermore, researchers have previously shown^64^ that the calculated theoretical internal quantum efficiency (IQE) increases 100-fold with a substantial reduction of threshold current density when the defect-limited carrier lifetime increased from 10 to 100 ns at 2.0% tensile strain and a doping of 5 × 10^18^ cm^–3^, thereby providing substantial motive for improvement in material quality. Along the same lines, our results indicate the prime quality of ε-Ge achievable via III–V metamorphic buffer heteroepitaxy and provide a pathway for the realization of QW heterostructures aimed toward developing Ge-based optical sources.

## 4 Conclusions

In summary, our work reports on the differences in structural crystallinity and carrier dynamics of ε-Ge grown on InGaAs and InAlAs metamorphic buffers and InGaAs/ε-Ge/InGaAs QW heterostructures. While symmetric strain relaxation and a smooth surface could be achieved on an InGaAs-based metamorphic buffer, asymmetric strain relaxation with a rough surface was observed in an InAlAs metamorphic buffer. High-resolution XRD analyses confirmed the efficacy of the metamorphic buffers in reducing the dislocation propagation to the active Ge layer. We did observe a discrepancy in estimation of the strain state of ε-Ge between XRD and Raman spectroscopy; nonetheless, successful transfer of tensile strain and pseudomorphic Ge epitaxy was confirmed in both heterostructures. Furthermore, surface morphology studied using AFM corresponds well to the XRD data, with higher symmetric relaxation showing a smoother surface morphology in ε-Ge grown on the InGaAs metamorphic buffer. Carrier dynamics studied using μ-PCD showed that the effective lifetime can be degraded due to increased surface roughness, corroborating the AFM analyses, and as such, InGaAs provides a better pathway for strained heteroepitaxy. Furthermore, we showed that the strain state of Ge in a QW heterostructure is almost unaltered after InGaAs overlayer growth, with minimal degradation of effective carrier lifetime, alluding to the prime quality of ε-Ge epilayers. These results bear practical implications for realization of strain-engineered Ge for the goal of realizing inter- and intrachip communications and strained Ge-based optical sources.

## Data Availability

The data that support the findings of this study are available from the corresponding author upon reasonable request.
